# Pressure Reactivity Index - Daily in Pediatric Traumatic Brain Injury

**DOI:** 10.21203/rs.3.rs-10461762/v1

**Published:** 2026-09-03

**Authors:** Suresh Nathan, Farooqi Ahmed, Michael Wolf, Prout Andrew, Ajit Sarnaik

**Affiliations:** Central Michigan University; Central Michigan University; Vanderbilt University Medical Center; Central Michigan University; Central Michigan University

**Keywords:** Cerebral Autoregulation, Neurocritical Care

## Abstract

**Purpose:**

Pressure Reactivity Index (PRx) is a measure of cerebral autoregulation that is associated with outcomes in pediatric traumatic brain injury. It is a resource-intensive tool that is not universally available. This study aims to assess the association of a novel low-resolution index derived from the same physiological inputs as the PRx, termed the Pressure Reactivity Index-Daily (PRx-D), with outcomes in children with traumatic brain injury (TBI).

**Methods:**

This is a secondary analysis of the Approaches and Decisions in Acute Pediatric Traumatic Brain Injury (ADAPT), an international multi-institutional comparative effectiveness trial that enrolled children from 2014–2017 who received intracranial monitoring for severe traumatic brain injury (Glasgow Coma Scale < 9). One thousand patients under 18 years of age were enrolled in the ADAPT trial. PRx-D was calculated using the Spearman correlation coefficient of hourly mean arterial pressures (MAP) and intracranial pressures (ICP). One PRx-D value was calculated for every day of admission for seven days. The minimum, median, maximum and day 1 PRx-D were used in the analysis. The primary outcome variable was dichotomized Glasgow Outcome Scale Extended Peds (DGOSE), with 1–4 defined as favorable outcome and 5–8 defined as unfavorable outcome. The secondary outcome variable was hospital death.

**Results:**

Higher values of median PRx-D, maximum PRx-D, and day 1 PRx-D were associated with unfavorable DGOSE in bivariate analysis. In multivariable analyses, higher maximum PRx-D (OR 2.45, 95% CI 1.25–4.82) was independently associated with unfavorable DGOSE. Higher median, minimum, maximum, and day 1 PRx-D were associated with hospital death in bivariate analysis. In multivariable analyses, higher minimum PRx-D, (OR 2.35, 95% CI 1.03–5.37) and median PRx-D (OR 4.34, 95% CI 1.42–13.23) were independently associated with hospital death.

**Conclusions:**

PRx-D, a clinical measure that can be obtained in lower resource settings, is independently associated with outcome in children with severe TBI.

## Introduction

More than one-fifth of children with severe traumatic brain injury (TBI) will die from their injuries and almost half will have a poor long term outcome(1,2). Evidence-based guidelines currently emphasize monitoring and treatment of pathologically increased intracranial pressure (ICP) while maintaining adequate cerebral perfusion pressure (CPP)(3). Although ICP monitoring is commonly employed in the management of severe traumatic brain injury (TBI), current guidelines provide weak evidence to support its use. Individual patients may have different ICP and CPP thresholds associated with mortality, indicating that a precision strategy tailored to individual physiology may improve care for children with severe TBI(4).

Real-time evaluation of cerebral autoregulation yields insight into an individual patient’s physiology, aiding in prognostication and treatment optimization(5). One measure of cerebral autoregulation is the pressure reactivity index (PRx), the correlation between mean arterial blood pressure (MAP) and intracranial pressure(6). Negative values for PRx occur in an individual patient when higher values of MAP are negatively associated with ICP, indicating intact cerebral autoregulation. When cerebral autoregulation is intact, pial arteriolar reflexive vasoconstriction from increased blood pressure results in cerebral blood volume reduction which reduces ICP(7,8). Recent work has demonstrated that lower PRx is independently associated with survival in children(9–13).

PRx calculation may provide value in caring for patients with TBI, but it is resource-intensive. It is calculated using a moving correlation coefficient between MAP and ICP averaged over a period of a few seconds(6). Prior authors have reported a PRx using lower frequency inputs that found similar associations with outcomes(14–17). However, these studies also use continuous processing of high-frequency vital sign data, limiting their applicability. Variations of the PRx using lower-frequency inputs may still yield clinically meaningful data. In this study, we evaluated PRx-Daily (PRx-D), a new, easily accessible clinical marker that uses routinely collected hourly MAP and ICP values. We hypothesized that a higher PRx-D would be associated with worse outcome in children with severe TBI.

## Methods

### Data collection

This is a secondary analysis of the Approaches and Decisions in Acute Pediatric TBI Trial (ADAPT). ADAPT was a comparative effectiveness study (NCT04077411) that enrolled 1000 patients admitted between 2014–2016 with severe TBI who underwent ICP monitor placement(18). Fifty-one centers from the US, UK, Spain, the Netherlands, India, South Africa, Australia, and New Zealand participated in this study(19). Patients under 18 years of age with severe TBI (defined as an initial post-resuscitation Glasgow Coma Scale [GCS] of less than 9) who had undergone ICP monitor placement at the enrolling institution were included in the ADAPT trial. Seven days of data following ICP monitor placement were collected. In the ADAPT trial, physiological variables such as ICP, MAP, and CPP were collected hourly for seven days following admission and enrollment. ICP monitoring was conducted via intraparenchymal catheters, intraventricular catheters, or both. Details regarding study protocols and characteristics of participant sites are described in previous publications(20,21). Local Institutional Review Board exempt status approval (“Low Resolution Pressure Reactivity Index in Pediatric TBI”, CMU 2023 – 614) was obtained on 9/29/2023 to acquire and perform analysis on the dataset. The deidentified data were obtained from the Federal Interagency Traumatic Brain Injury Research Informatics System. All procedures were in accordance with the ethical standards of the CMU IRB and the Helsinki Declaration of 1975.

We collected basic demographic variables and injury characteristics including age, race, sex, ethnicity, and GCS. GCS was stratified into a tripartite classification (3, 4–5, 6–8) as previously reported(22). Abusive head trauma (AHT) was defined as present if classified as possible, probable, or definite abuse in the primary trial. Pre-hospital cardiac arrest, hypotension, hypoxia, and seizure were also included as per the primary trial. Hourly CPP and ICP, as obtained in the ADAPT trial, were used in the analysis and calculation of PRx-D. Median CPP and median ICP over the entire study period for each patient were also used as covariates in the analysis. The PRx-D was calculated for each day using Spearman’s correlation coefficient. The Spearman’s correlation coefficient was used because the data did not follow a normal distribution. PRx-D was calculated using 24 hourly values of ICP and MAP. The use of hourly values reflects conventional charting in intensive care units and the pre-existing data structure of the ADAPT trial. To ensure the robustness of the correlation coefficient calculation while maximizing the number of eligible PRx-D patient-days included in the analysis, we established a threshold of requiring at least 10 pairs of hourly data points (i.e., no more than 14 pairs of missing values) for a PRx-D calculation. Missing value imputation was not performed. The Python library *pandas* (version 2.2.2) was utilized for the calculation of PRx-D(23). For each patient, a discrete PRx-D value was generated for each day of intracranial monitoring, up to seven days. From this set of daily values, we derived the primary predictor variables: median PRx-D, minimum PRx-D, maximum PRx-D, as well as the value from the first 24-hour period (day 1 PRx-D). The primary outcome variable was dichotomized Glasgow Outcome Scale Extended Peds (DGOSE). The full Pediatric Glasgow Outcome Scale used in the ADAPT trial is as follows: (8 – Death; 7 – Vegetative State; 6 – Lower Severe Disability; 5 – Upper Severe Disability; 4 – Lower Moderate Disability; 3 – Upper Moderate Disability; 2 – Lower Good Recovery; 1 – Upper Good Recovery) (24). GOSE Peds 1–4 was defined as favorable outcome and 5–8 was defined as unfavorable outcome. The secondary outcome was hospital death. As an exploratory analysis, individual patient trends of PRx-D, hourly ICP, and hourly CPP were graphed and compared side-by-side. Patients whose PRx-D values were close to the median in the survival, non-survival, unfavorable DGOSE, and favorable DGOSE groups were compared.

### Statistical Analysis

We used non-parametric tests in this study because the physiological data, tested using the Kolmogorov-Smirnov test, were not normally distributed. The associations of the median, day 1, minimum, and maximum PRx-D over seven days with hospital death and DGOSE were assessed using Wilcoxon rank-sum tests. All variables which had a p value < 0.2 in the bivariate analyses as well as age, race, and sex were included in the multivariable analyses. Multivariable binary logistic regression models were created to analyze the relationship of the PRx-D metrics with hospital death and DGOSE, with each measurement evaluated in its own model to remove the effect of collinearity between the various PRx-D metrics. A sensitivity analysis was performed using minimum, median, and maximum PRx-D over the first three days of admission. Alpha was set at 0.05. SAS version 9.4 (SAS Institute Inc., Cary, NC, USA) was used for the analysis. R version 4.4.1 was used for obtaining Receiver Operator Characteristic (ROC) curves and the sensitivity analysis binary logistic regression (R Foundation for Statistical Computing, Vienna, Austria.) The pROC package version 1.18.5 in R was used to obtain the ROC curves(25). Youden’s J statistic was used to obtain the optimal cutoff threshold.

## Results

In the overall cohort, there were 835 survivors (83.5%) and 165 non-survivors (16.5%). A total of 336 patients (33.6%) had a GCS of 3, 162 patients had a GCS of 4–5 (16.2%), and 502 patients (50.2%) had a GCS of 6–8. Of the 812 patients who had GOSE measured, 325 patients (40%) had a favorable DGOSE, and 487 patients (60%) had an unfavorable DGOSE, including 180 (22%) who died. Demographic data are summarized in Table 1.

Eleven patients did not have a PRx-D calculation due to insufficient data. On average, the PRx-D was highest on the second day of hospitalization for all patients. Daily distributions of PRx-D data for all the patients are contained in Supplemental Figure 1 and Supplemental Table 1. In the entire cohort, day 2 had the highest median PRx-D value. Supplemental Figure 2 illustrates the frequency distribution of missing values across days with available PRx-D calculations. Most patients were missing zero to two values in the PRx-D calculations.

### DGOSE

In bivariate analysis, admission tripartite GCS, abuse, cardiac arrest, hypotension, hypoxia, day one PRx-D, median PRx-D, maximum PRx-D, median ICP, and median CPP were significantly different in patients with unfavorable DGOSE compared to those with favorable DGOSE (Table 2 and Figure 1). In multivariable analyses, higher maximum PRx-D (OR 2.45, 95% CI 1.25 – 4.82) was independently associated with unfavorable DGOSE. Figure 2 contains odds ratios and confidence intervals from the multivariable model for all PRx-D metrics. Median CPP was not significantly associated with DGOSE in the multivariable analyses. The complete results of the multivariable analyses are in Table 3. Maximum PRx-D had an area under the receiver operator characteristic curve (AUROC) of 0.62 (95% CI 0.59 – 0.66) with an optimal cutoff of 0.59. Supplemental Figure 3 contains AUROC values for all PRx-D metrics as a discriminator of DGOSE. ICP, CPP, and PRx-D trends are graphed in a patient with an unfavorable DGOSE and a patient with a favorable DGOSE in Supplemental Figure 4.

### Hospital Death

In bivariate analysis, non-survivors had significantly higher rates of abuse, cardiac arrest, hypotension, hypoxia, all PRx-D metrics, and median ICP compared to survivors. Non-survivors had significantly lower tripartite GCS and median CPP compared to survivors (Table 4, Figure 3). In multivariable analyses, higher minimum PRx-D (OR 2.35, 95% CI 1.03 – 5.37) and median PRx-D (OR 4.34, 95% CI 1.42 – 13.23) were independently associated with hospital death (Figure 4). The full multivariable analyses can be seen in Table 5. Median PRx-D had an AUROC of 0.75 (95% CI 0.7 – 0.8) with an optimal cutoff of 0.24. Minimum PRx-D had an AUROC of 0.73 (95% CI 0.68 – 0.78) with an optimal cutoff of 0.06 (Supplemental Figure 5). A survivor and non-survivor patient trend with ICP, CPP and PRx-D is in Supplemental Figure 6.

### 3-Day PRx-D Sensitivity Analysis

3-Day Maximum PRx-D (OR 1.98, 95% CI 1.04 – 3.78) was independently associated with unfavorable DGOSE in multivariable logistic regression analysis (Supplemental Table 2). 3-Day Median PRx-D and 3-Day Minimum PRx-D were not significantly associated with unfavorable DGOSE in multivariable logistic regression analyses (Supplemental Table 3 & 4). 3-Day Median PRx-D (OR 2.96, 95% CI 1.15 – 7.62) and 3-Day Maximum PRx-D (OR 2.68, 95% CI 1.05 – 6.84) were independently associated with hospital death in multivariable logistic regression analyses (Supplemental Table 5 & 6). 3-Day Minimum PRx-D was not significantly associated with hospital death in the multivariable analysis (Supplemental Table 7). AUROC values for the 3-Day PRx-D metrics are in Supplemental Figures 3 and 5.

## Discussion

In this secondary analysis of the ADAPT study, various PRx-D metrics were associated with worsened DGOSE and hospital death even after adjusting for other clinical variables known to be predictive of outcome. Maximum PRx-D was independently associated with unfavorable DGOSE after adjusting for injury severity factors. In multivariable analysis, minimum PRx-D and median PRx-D were independently associated with hospital death. A key finding of this study is that PRx-D appears to provide prognostic information that is distinct from that offered by ICP alone. For instance, in bivariate analysis comparing favorable and unfavorable DGOSE, median ICP, while statistically significantly different between both groups, only differed by 2 mmHg, a margin unlikely to be clinically significant by itself. Additionally, median CPP was not independently associated with DGOSE. Adding to the cornerstone of ICP and CPP measurements in acute TBI management, PRx-D offers a complementary layer of information that our data suggests can help with prognostication.

In the ROC analysis for maximum PRx-D, the optimal cut-off for unfavorable DGOSE was 0.59 which is higher than previously reported values(9,13,26). This difference likely comes from using a maximum value for PRx-D while previous studies have used average values. With an AUROC of 0.62, the utility of maximum PRx-D as an independent discriminatory tool for DGOSE is limited. Consistent with other studies, we found that a threshold of 0.24 for median PRx-D optimally differentiated mortality(10,27–29). In the ROC analysis, PRx-D was a better discriminator of survival than DGOSE, and the sensitivity analysis indicates that this can be assessed after three days of admission. The robust discriminatory ability and independent association of median PRx-D and death was also seen in the 3-day sensitivity analysis, highlighting its value for early mortality prediction. Additionally, maximum 3-Day PRx-D provides prognostic value early in the hospital course for unfavorable DGOSE.

Our study demonstrates associations of PRx-D with outcomes in pediatric severe TBI. Its underlying physiological underpinnings are likely not the same as the PRx. Cerebral autoregulation captured by PRx works on a vastly different timescale than that captured by PRx-D(30). To capture the fast timescale of autoregulation, PRx uses data obtained from continuous signal processing, incorporating fluctuations in ICP that occur over seconds and minutes(31,32). The effect of these fluctuations from the “biological oscillator” are necessary to comprehensively assess autoregulation as estimated by PRx(6). Autoregulation insights using PRx cannot be obtained at the bedside without resource-intensive high-frequency data input. PRx-D, by its nature of hourly data input, is not designed to capture the high-frequency components of autoregulation. Consequently, PRx-D cannot represent minute-scale cerebrovascular dynamics. Trends in PRx-D are likely affected by physiological processes that affect systemic and intracranial pressure over longer durations such as changes in intracranial compliance, variations in sympathetic tone over hours, or disruptions to physiologic diurnal changes in cerebral blood flow(33). As previously demonstrated with a minute-by-minute sample derived pressure reactivity index, ICP waves with periods less than two minutes may not be necessary for estimation of autoregulation and prognostication and may benefit from reduction of noise(34). This could explain why both lower and higher frequency PRx measures have been linked to outcomes in patients with brain injuries(35). It remains possible that PRx-D may capture a slow component of autoregulation, which, while lacking information about the fast component of autoregulation, represents one part of the overall autoregulatory process. In interpreting the meaning of PRx-D, one must be careful not to conflate it with traditional concepts of cerebral autoregulation which occur at short time scales. As previously mentioned, other physiologic mechanisms should be considered. Ultimately, our findings show that it is an independent predictor of functional outcome and mortality in this pediatric TBI cohort.

Although the present study calculates PRx-D using the Spearman correlation coefficient instead of the traditionally used Pearson correlation coefficient due to this study’s non-normally distributed data, we show similar associations with death and DGOSE to previous reports(10,11,36,37). PRx-D, in comparison to PRx, uses data that occurs at a larger time scale, increasing its likelihood of exposure to larger blood pressure ranges and their associated non-linear relationships with ICP. This issue does not apply to PRx with its much faster calculation. Thus, the non-parametric Spearman correlation coefficient was chosen as a more appropriate measure for PRx-D. Prior authors have reported a significant association of ultra low-resolution PRx in children (using 30-min time windows) with outcome and death, though their use of processed continuous data limits availability in many institutions(16,17). Using data that is already routinely collected in the intensive care setting confers an advantage to PRx-D as it allows its use in all institutions. In the future, PRx-D could be feasibly implemented through electronic medical record toolkits using pre-existing charted data, streamlining its use. The utilization of PRx-D may contribute to the risk-benefit analysis of pursuing ICP monitoring, a decision challenged by risks and prior studies questioning its utility(38,39). As part of a multi-modal monitoring strategy, PRx-D can help clinicians gauge severity of injury and trajectory of illness. Specifically in patients that are toward the end of the acute post-trauma period, PRx-D can add additional data that informs the decision to continue strict neuroprotective measures or de-escalate treatment in patients with a difficult clinical examination.

There are several limitations to this study. One limitation lies in the inherent nature of calculating PRx-D. Since hourly data points are used, fluctuations in ICP and MAP that occur on the order of seconds to minutes are not captured by PRx-D. While these fluctuations may not be necessary to obtain useful prognostication information, it complicates the interpretation of PRx-D as an index of autoregulation (34). Due to the long timescale of data inputs, PRx-D is unable to calculate an optimal cerebral perfusion pressure. Additionally, median CPP and ICP datapoints were used in the analysis, which also excludes the effects of shorter-term fluctuations in ICP and MAP. Another limitation is missing MAP and ICP values that reduce the fidelity of PRx-D calculations. Missing value imputation was not performed because it would add an additional layer of complexity in the calculation of PRx-D which would limit its accessibility. As seen in Supplemental Fig. 2, most calculated PRx-D values were missing zero to two values. Given the high-degree of completeness of the data involved in PRx-D calculation, we anticipate that the effect of missing values is minimized. Due to death, varying length of stays, and missing data, not all patients had seven days of data. This limitation was addressed with the 3-day PRx-D sensitivity analysis which ameliorated potential bias from varying monitoring durations, such as in patients with shorter ICU stays due to early death, early ICP monitoring discontinuation, or other factors.

This study uses a novel index, easily calculated with routinely collected data, that is predictive of outcome in this cohort of children with severe traumatic brain injury. Our findings suggest that PRx-D potentially adds complementary information to standard ICP monitoring. To clarify whether PRx-D could represent a purported slow frequency component of autoregulation, a future study comparing PRx-D to various low-resolution PRx calculations derived from continuous data would be valuable. Comparing the associations of the PRx-D and the PRx with outcome measures in a prospective pediatric TBI cohort would help elucidate the value of PRx-D for prognostication in comparison with the PRx. PRx-D can provide an incremental prognostic value when adding it to other variables in established prognostic models, which can be pursued in future studies. Prospective studies that assess the influence of PRx-D on clinical decision support will further help determine its potential utility as a bedside tool.

## Conclusion

A low-resolution version of the pressure reactivity index, PRx-D, is significantly and independently associated with dichotomized Glasgow Outcome Scale – Extended and hospital death in pediatric TBI patients. This tool has potential for low-cost implementation to provide prognostication information for children with severe TBI.

## Supplementary Files

This is a list of supplementary files associated with this preprint. Click to download.

- Supplement72026.docx

## Figures and Tables

**Figure 1:**
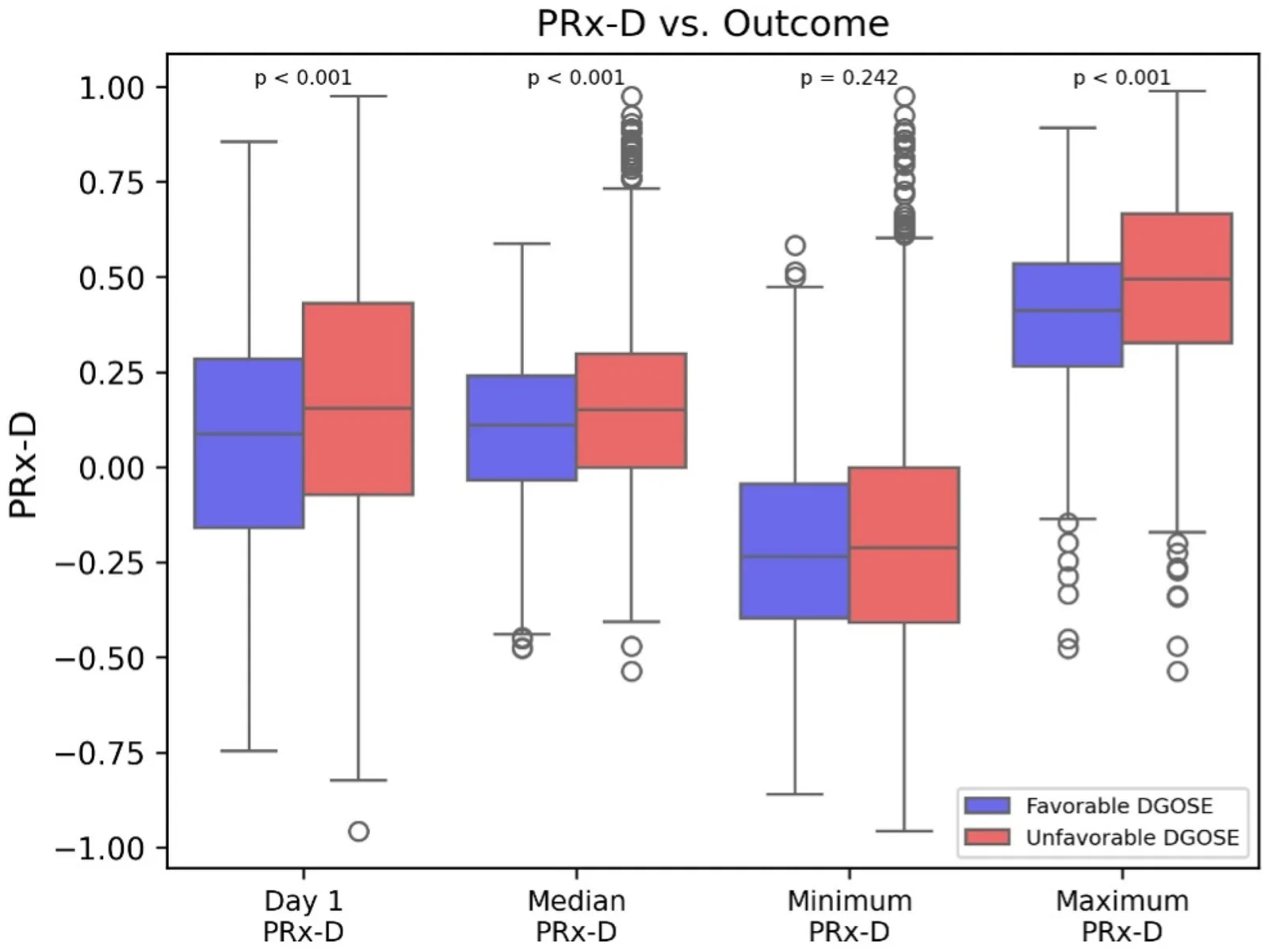
Legend not included with this version.

**Figure 2:**
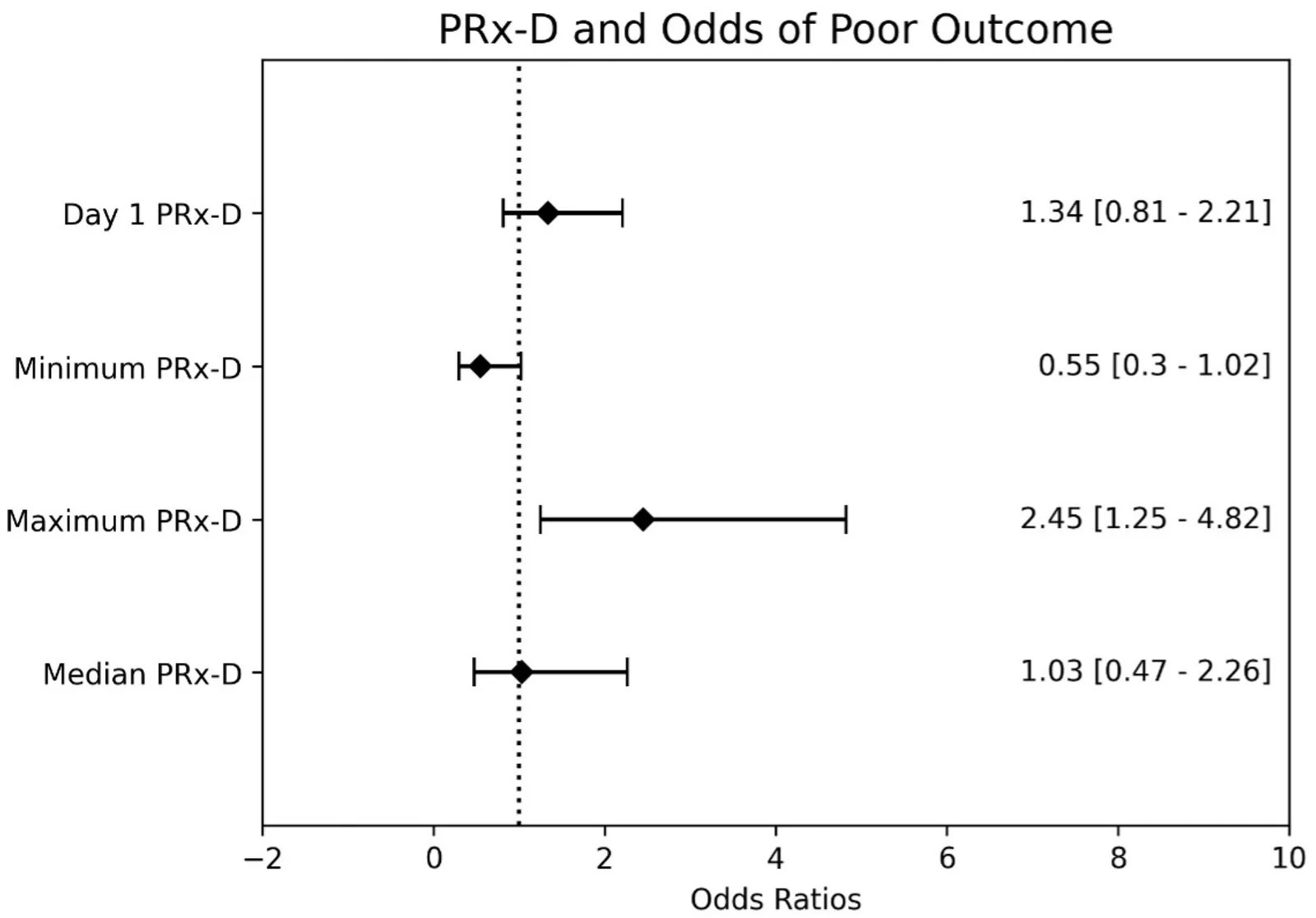
Legend not included with this version.

**Figure 3:**
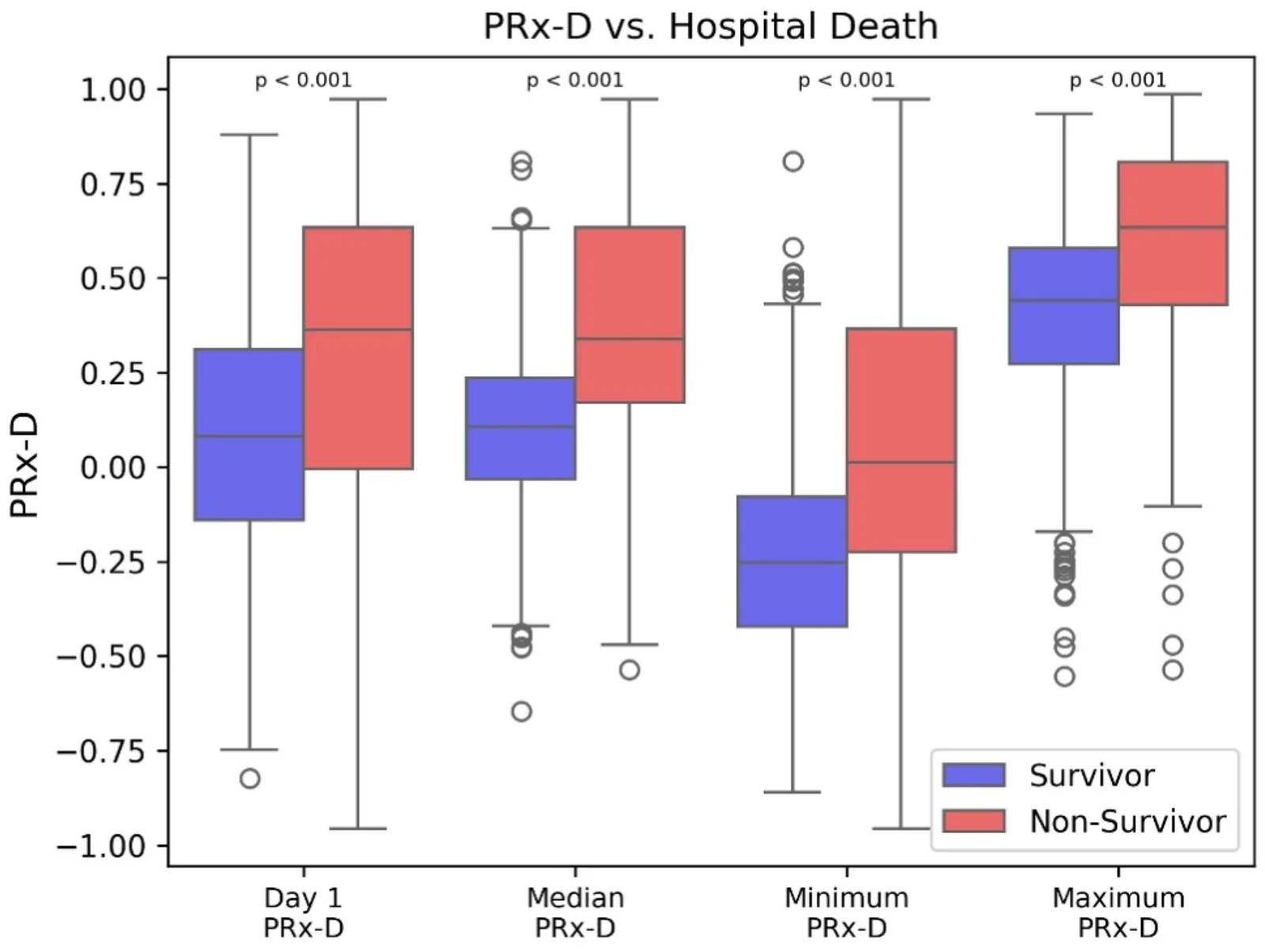
Legend not included with this version.

**Figure 4:**
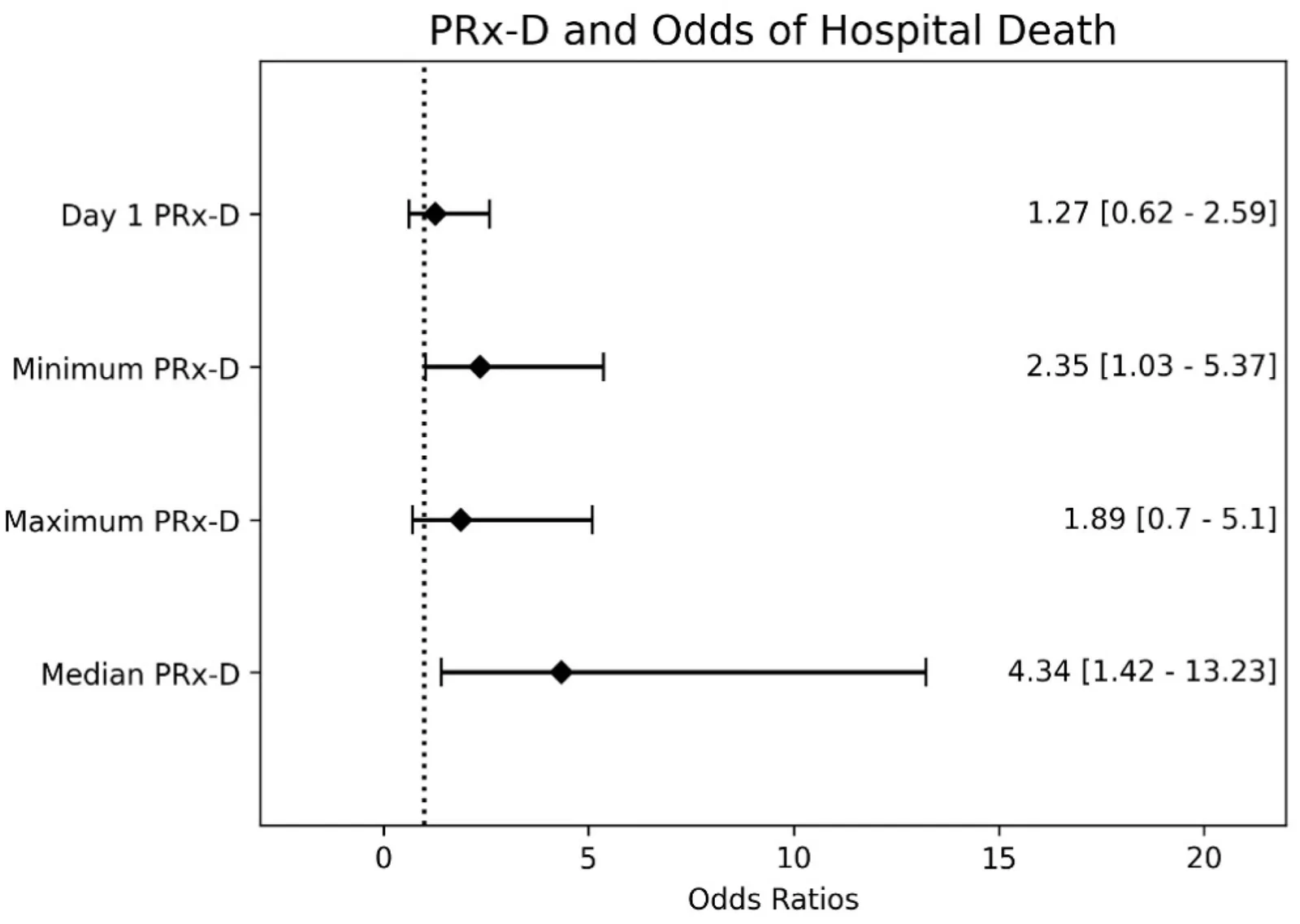
Legend not included with this version.

**Table 1.** Characteristics of All Patients (n = 1000)

Variable	n (%) or Median*
Age (years)	6 (2 – 12)
Sex (M)	645 (64.5)
Race	s
Asian	113 (11.3)
Black	213 (21.3)
Other	113 (11.3)
White	561 (56.1)
GCS	
3	336 (33.6)
4–5	168 (16.8)
6–8	502 (50.2)
Abuse	178 (17.8)
Cardiac Arrest	33 (3.3)
Hypotension	254 (25.4)
Hypoxia	49 (4.9)
Seizure	109 (10.9)

Day One PRx-D	0.11 (−0.12 – 0.36)
Minimum PRx-D	−0.22 (−0.4 – −0.04)
Median PRx-D	0.13 (−0.02 – 0.27)
Maximum PRx-D	0.46 (0.28 – 0.61)
Median CPP	64 (57 – 71)
Median ICP	13 (9 – 16)

Death	165 (16.5)
Unfavorable DGOSE	487 (60)
Length of Stay (days)	10 (5 – 16)

* Median (25^th^ percentile – 75^th^ percentile)

**Table 2.** Characteristics of Patients with Favorable GOS–E vs. Unfavorable GOS–E

Variable	Good outcome (n = 325)	Bad outcome (n = 487)	p Value
Age	6 (3 – 12)	6 (2 – 12)	0.959
Sex (M)	215 (66.15)	307 (63.04)	0.405
Race			
Asian	62 (59.05)	43 (40.95)	< 0.001
Black	52 (34.44)	99 (65.56)	
Other	29 (32.22)	61 (67.78)	
White	182 (39.06)	284 (60.94)	
GCS ^a^			
3	43 (26.84)	199 (73.16)	< 0.001
4–5	38 (28.15)	97 (71.85)	
6–8	214 (52.84)	191 (47.16)	
Abuse	26 (8.0)	102 (20.9)	< 0.001
Cardiac Arrest	3 (0.92)	25 (5.13)	0.001
Hypotension	59 (18.15)	152 (31.21)	< 0.001
Hypoxia	10 (3.08)	30 (6.16)	0.047
Seizure	35 (10.77)	41 (8.42)	0.260

Day one PRx-D	0.088 (− 0.16 – 0.28)	0.15 (− 0.07 – 0.43)	< 0.001
Minimum PRx-D	−0.24 (−0.40 – 0.05)	−0.21 (−0.41 – 0)	0.242
Median PRx-D	0.11 (− 0.03 – 0.24)	0.15 (0 – 0.30)	< 0.001
Maximum PRx-D	0.41 (0.26 – 0.54)	0.49 (0.33 – 0.67)	< 0.001
Median CPP	66.25 (60 – 71.38)	63 (53 – 69)	< 0.001
Median ICP	12 (9 – 15)	14 (10 – 20)	< 0.001

n (%) or Median (25^th^ percentile – 75^th^ percentile)

**Table 3.** Multivariable Binary Logistic Regression PRx-D vs. Dichotomized GOSE

	Odds Ratios (95% Confidence Intervals)			
Variables	Day 1 PRx-D	Minimum PRx-D	Maximum PRx-D	Median PRx-D
Age (years)	1.01 (0.97 – 1.05)	1.01 (0.97 – 1.05)	1.01 (0.97 – 1.05)	1.01 (0.97 – 1.01)
Sex (M)	1.16 (0.84 – 1.61)	1.17 (0.84 – 1.62)	1.17 (0.85 – 1.63)	1.16 (0.84 – 1.61)
Race				
Asian vs. White	0.66 (0.41 – 1.09)	0.66 (0.40 – 1.08)	0.65 (0.40 – 1.06)	0.64 (0.39 – 1.04)
Black vs. White	1.01 (0.71 – 1.71)	1.10 (0.71 – 1.69)	1.06 (0.69 – 1.65)	1.10 (0.71 – 1.69)
Other vs. White	1.51 (0.89 – 2.57)	1.48 (0.87 – 2.51)	1.50 (0.88 – 2.55)	1.53 (0.90 – 2.58)
GCS				
3 vs. 6–8	2.08 (1.43 – 3.01)	2.06 (1.43 – 2.98)	2.02 (1.39 – 2.92)	2.05 (1.42 – 2.96)
4–5 vs. 6–8	2.14 (1.35 – 3.39)	2.18 (1.38 – 3.45)	2.10 (1.32 – 3.33)	2.22 (1.40 – 3.50)
Abuse	2.79 (1.59 – 4.90)	2.65 (1.52 – 4.61)	2.55 (1.46 – 4.45)	2.63 (1.51 – 4.59)
Cardiac Arrest	2.62 (0.72 – 9.59)	2.90 (0.79 – 10.68)	2.57 (0.71 – 9.35)	2.73 (0.75 – 9.95)
Hypotension	1.51 (1.03 – 2.23)	1.44 (0.98 – 2.11)	1.43 (0.97 – 2.10)	1.46 (0.99 – 2.14)
Hypoxia	1.48 (0.64 – 3.45)	1.53 (0.66 – 3.54)	1.57 (0.67 – 3.64)	1.53 (0.66 – 3.54)
Seizure	1.35 (0.78 – 2.32)	0.70 (0.40 – 1.20)	0.71 (0.41 – 1.22)	0.70 (0.41 – 1.21)
Day 1 PRx-D	1.34 (0.81 – 2.21)			
Minimum PRx-D		0.55 (0.30 – 1.02)		
Maximum PRx-D			2.45 (1.25 – 4.82)	
Median PRx-D				1.03 (0.47 – 2.26)
Median CPP (mmHg)	1.00 (0.98 – 1.03)	1.00 (0.98 – 1.02)	1.00 (0.98 – 1.02)	1.00 (0.98 – 1.02)
Median ICP (mmHg)	1.06 (1.03 – 1.09)	1.06 (1.03 – 1.09)	1.06 (1.03 – 1.09)	1.06 (1.03 – 1.09)

**Table 4.** Characteristics of Survivors vs. Non–Survivors

Variable	Survivor (n = 835)	Non-Survivor (n = 165)	p Value
Age (years)	7 (3 – 12)	5 (1 – 12)	0.079
Sex (M)	538 (64.43)	107 (64.82)	0.99
Race			
Asian	100 (88.5)	13 (11.5)	0.486
Black	178 (83.57)	35 (16.43)	
Other	94 (83.19)	19 (16.81)	
White	463 (82.53)	98 (17.47)	
GCS			
3	241 (71.73)	95 (28.27)	< 0.001
4–5	126 (77.78)	36 (22.22)	
6–8	468 (93.23)	34 (6.77)	
Abuse	131 (13.1)	47 (28.5)	< 0.001
Cardiac Arrest	15 (1.8)	18 (10.91)	< 0.001
Hypotension	195 (23.35)	59 (35.76)	< 0.001
Hypoxia	34 (4.07)	15 (9.09)	0.006
Seizure	98 (11.74)	11 (6.67)	0.056
Day one PRx-D	0.08 [− 0.14 – 0.31]	0.363 [− 0.01 – 0.63]	< 0.001
Minimum PRx-D	− 0.25 [− 0.42 – 0.08]	0.0130 [−0.23 – 0.37]	< 0.001
Median PRx-D	0.11 [− 0.31 – 0.24]	0.338 [0.17 – 0.64]	< 0.001
Maximum PRx-D	0.44 [ 0.27 – 0.58]	0.633 [0.43 – 0.81]	< 0.001
Median CPP (mmHg)	65 [59.9 – 72]	45 [15.75 – 61.5]	< 0.001
Median ICP (mmHg)	12 [9 – 15]	28 [15 – 61.25]	< 0.001

Categorical variables show percentages within the survivor or within the non-survivor group. Continuous variables are median (25^th^ percentile – 75^th^ percentile).

**Table 5.** Multivariable Binary Logistic Regression PRx-D vs. Hospital Death

	Odds Ratios (95% Confidence Intervals)			
Variable	Day 1 PRx-D	Minimum PRx-D	Maximum PRx-D	Median PRx-D
Age	1.04 (0.98 – 1.10)	1.03 (0.97 – 1.09)	1.03 (0.97 – 1.09)	1.03 (0.97 – 1.09)
Sex (F)	0.94 (0.57 – 1.54)	0.90 (0.55 – 1.47)	0.92 (0.56 – 1.51)	0.91 (0.56 – 1.49)
Race				
Asian vs. White	1.09 (0.47 – 2.53)	0.96 (0.41 – 2.26)	1.07 (0.46 – 2.46)	1.02 (0.44 – 2.37)
Black vs. White	0.92 (0.48 – 1.73)	0.90 (0.47 – 1.67)	0.84 (0.44 – 1.59)	0.85 (0.45 – 1.61)
Other vs. White	0.98 (0.45 – 2.12)	0.99 (0.45 – 2.14)	0.93 (0.43 – 2.02)	0.97 (0.44 – 2.11)
GCS				
3 vs. 6–8	3.12 (1.80 – 5.41)	3.10 (1.79 – 5.38)	3.13 (1.80 – 5.42)	3.06 (1.76 – 5.31)
4–5 vs 6–8	2.33 (1.19 – 4.57)	2.48 (1.27 – 4.83)	2.41 (1.24 – 4.71)	2.38 (1.22 – 4.64)
Abuse	1.12 (0.55 – 2.26)	1.08 (0.54 – 2.19)	1.02 (0.50 – 2.07)	1.01 (0.50 – 2.06)
Cardiac Arrest	7.62 (2.69 – 21.58)	7.09 (2.52 – 19.99)	7.33 (2.59 – 20.72)	7.22 (2.55 – 20.42)
Hypotension	0.70 (0.39 – 1.27)	0.70 (0.39 – 1.27)	0.68 (0.38 – 1.24)	0.68 (0.37 – 1.23)
Hypoxia	1.27 (0.44 – 3.64)	1.26 (0.44 – 3.64)	1.27 (0.45 – 3.60)	1.23 (0.43 – 3.21)
Seizure	0.70 (0.30 – 1.60)	0.70 (0.31 – 1.61)	0.67 (0.29 – 1.55)	0.69 (0.30 – 1.6)
Day 1 PRx-D	1.27 (0.62 – 2.59)			
Minimum PRx-D		2.35 (1.03 – 5.37)		
Maximum PRx-D			1.89 (0.70 – 5.10)	
Median PRx-D				4.34 (1.42 – 13.23)
Median CPP (mmHg)	0.95 (0.92 – 0.98)	0.96 (0.93 – 0.99)	0.96 (0.93 – 0.99)	0.96 (0.93 – 0.99)
Median ICP (mmHg)	1.07 (1.04 – 1.11)	1.07 (1.04 – 1.10)	1.07 (1.04 – 1.11)	1.07 (1.03 – 1.10)

## Data Availability

This study is a secondary analysis of the ADAPT trial (NCT04077411). Local Institutional Review Board exempt status approval (“Low Resolution Pressure Reactivity Index in Pediatric TBI”, CMU 2023–614) was obtained on 9/29/2023 to acquire and perform analysis on the dataset. The ADAPT de-identified data used in this study is available on FITBIR (https://fitbir.nih.gov/) and was obtained through a data access request.
